# Single cell analysis of the localization of the hematopoietic stem cells within the bone marrow architecture identifies niche-specific proliferation dynamics

**DOI:** 10.3389/fmed.2023.1166758

**Published:** 2023-04-28

**Authors:** Maria Mazzarini, Francesca Arciprete, Orietta Picconi, Mauro Valeri, Paola Verachi, Fabrizio Martelli, Anna Rita Migliaccio, Mario Falchi, Maria Zingariello

**Affiliations:** ^1^Department of Biomedical and Neuromotor Sciences, University of Bologna, Bologna, Italy; ^2^Altius Institute for Biomedical Sciences, Seattle, WA, United States; ^3^Unit of Microscopic and Ultrastructural Anatomy, University Campus Bio-Medico, Rome, Italy; ^4^National Center for HIV/AIDS Research, Istituto Superiore di Sanità, Rome, Italy; ^5^Center for Animal Experimentation and Well-Being, Istituto Superiore di Sanità, Rome, Italy; ^6^National Center for Preclinical and Clinical Research and Evaluation of Pharmaceutical Drugs, Istituto Superiore di Sanità, Rome, Italy

**Keywords:** hematopoietic stem cells, GFP reporter, quantitative microscopy, microenvironment, aging, adipocytes

## Abstract

**Introduction:**

Hematopoietic stem cells (HSC) reside in the bone marrow (BM) in specialized niches which provide support for their self-replication and differentiation into the blood cells. Recently, numerous studies using sophisticated molecular and microscopic technology have provided snap-shots information on the identity of the BM niches in mice. In adults, HSC are localized around arterioles and sinusoids/venules whereas in juvenile mice they are in close to the osteoblasts. However, although it is well recognized that in mice the nature of the hematopoietic niche change with age or after exposure to inflammatory insults, much work remains to be done to identify changes occurring under these conditions. The dynamic changes occurring in niche/HSC interactions as HSC enter into cycle are also poorly defined.

**Methods:**

We exploit mice harboring the *hCD34tTA/Tet-O-H2BGFP* transgene to establish the feasibility to assess interactions of the HSC with their niche as they cycle. In this model, *H2BGFP* expression is driven by the TET trans-activator under the control of the human *CD34* promoter which in mice is active only in the HSC. Since Doxycycline inhibits TET, HSC exposed to this drug no longer express *H2BGFP* and loose half of their label every division allowing establishing the dynamics of their first 1-3 divisions. To this aim, we first validated user-friendly confocal microscopy methods to determine HSC divisions by hemi-decrement changes in levels of GFP expression. We then tracked the interaction occurring in old mice between the HSC and their niche during the first HSC divisions.

**Results:**

We determined that in old mice, most of the HSC are located around vessels, both arterioles which sustain quiescence and self-replication, and venules/sinusoids, which sustain differentiation. After just 1 week of exposure to Doxycycline, great numbers of the HSC around the venules lost most of their GFP label, indicating that they had cycled. By contrast, the few HSC surrounding the arterioles retained maximal levels of GFP expression, indicating that they are either dormant or cycle at very low rates.

**Conclusion:**

These results reveal that in old mice, HSC cycle very dynamically and are biased toward interactions with the niche that instructs them to differentiate.

## 1 Introduction

Recent studies, using a combination of mouse models, expression profiling and sophisticated multicolor confocal microscopy coupled with high power computer technologies, have demonstrated that under steady-state conditions the hematopoietic stem cells (HSC) are localized in areas of the bone marrow (BM) microenvironment defined as the HSC niche (1–5). The BM contains several niches, each one of them representing a unique cellular configuration that regulates specific aspects of the HSC fate. The most studied of the BM niches are the endosteal and the vascular niche. The endosteal niche consists of osteoclasts, osteoblasts and mesenchymal cells and is supposed to retain HSC into quiescence and to assure that they undergo self-renewal upon division. The vascular niche is composed by the endothelial cells surrounding the vessels and by pericytes surrounding the sinusoids and regulates the differentiation and mobilization of the HSC. The niche may affect HSC fate directly, by secreting factors such as stem cell factor (SCF) and C-X-C motif chemokine ligand 12 (CXCL12), necessary for their survival and proliferation, and indirectly, by recruiting cells such as megakaryocytes, macrophages and other stromal cells, which are responsible to secrete factors, such as platelet factor 4 (PF-4, also known as CXCL4) and Transforming Growth Factor-β (TGF-β) that induce HSC into quiescence allowing them to retain stemness (6).

In adult mice and under steady state conditions, the majority of HSC are located near the vascular niche, in particular around the arterioles (7). However, the location of HSC withing the BM architecture changes with age and under conditions of inflammation. In juvenile mice (3-weeks of age) HSC are found in high numbers near stromal cells expressing CXCL12 or associated with the osteoblast niche of the bone. By contrast, studies in old (>8-months old) mice, have identified profound changes in the localization of the HSC within the BM architecture, although the identity of the niche in these old mice has not been well characterized as yet (8–11). Experimentally induced inflammation, such as treatment with the pro-inflammatory cytokine interferon-γ, greatly reduces the interaction of the HSC with the arteriole niche, reducing their self-renewal potential, while increasing their interaction with the perisinusoidal niche, favoring differentiation and exhaustion (7, 12–14).

All these studies have two caveats: (1) they are all conducted on mice with the same genetic background (C57Bl6) and therefore they do not reflect the variability of the human population and (2) they provide snapshots of HSC fate in the mouse BM but say little on the dynamics of HSC location as they cycle. To study the cycling of the HSC, the Moore laboratory has developed the *hCD34tTA/TET-O-H2BGFP* transgenic mouse model (15–17). The *hCD34tTA* gene encodes a tet-Transactivator (tTA), which is suppressed by doxycycline (Doxy), under the control of the human CD34 promoter. The human CD34 promoter is active only in the HSC, restraining the expression of tTA to the HSC (16). *TET-O-H2B-GFP* encodes a H2B-GFP fusion gene under the control of the TetO element activated by tTA which is expressed only in the HSC. Therefore, in double *hCD34tTA/TET-O-H2BGFP* transgenic mice only the HSC are labeled by GFP. When mice are treated with Doxy, HSC lose half of their label following each division. It takes approximately 4 divisions for the HSC to lose their label. By flow cytometry, the HSC, identified by the SLAM phenotype (18, 19), may be divided based on hemi-decrements of GFP intensity into four populations: G0, which express maximal GFP levels and never divided, and cells which divided 1, 2, 3, and 4 times expressing, respectively, half (GFP1), a quarter (GFP2), an eighth (GFP3) or none (GFP4) of the maximal GFP level. Using this model, the Moore laboratory has demonstrated the feasibility to study the dynamics of the HSC proliferation in young mice under steady state conditions and after stimulation with G-SCF (20–23). By tracking the cumulative division history of the HSC throughout life, the Moore laboratory has also identified a slow-cycling HSC population that contains all the long-term repopulation activity of the HSC (15). This population undergoes four self-renewal divisions which last progressively longer time and then enters in a state of dormancy which is retained for the rest of the life of the mice. The niche which sustains the “dormant” and “cycling” HSC in these old mice has not been identified as yet.

Since the *hCD34tTA/TET-O-H2BGFP* transgenic mice express autofluorescence signals only in the bones (Dr. Moore personal communication), we decided to exploit the power of confocal microscopy to validate these mice as a model to track the HSC localization within the BM architecture as they divide. Given the limited information available on the identity of the niche which sustains the “dormant” and the “cycling” HSC in old mice discussed above (15), these studies were performed in *hCD34tTA/TET-O-H2BGFP* mice >11-months old. In addition, since TGF-β is one of the factors which retain the HSC into quiescence (24), the *hCD34tTA/TET-O-H2BGFP* mutation was brought in the CD1 background which express a baseline pro-inflammatory signature (25) that includes high levels of TGF-β. Th CD1 model allow assessing the number of HSC in proliferation under physiologically high TGF-β levels similar to that found in some of the elder population (26).

## 2 Materials and methods

### 2.1 Mice

Transgenic mice were bred in the animal facility of Istituto Superiore di Sanità as described (27, 28). The original *huCD34tTA and TetO-H2BGFP* single transgenic mice were provided by Dr. Katery Moore (15–17). The single mutant mice were bread with wild type CD1 mice to create double *huCD34tTA/TetO-H2BGFP* transgenic mice in the animal facility of Istituto Superiore di Sanità according to standard genetic protocols (15–17) and backcrossed for at least 10 generation before being included in this study. All the mice used in these experiments were genotyped by PCR at birth as a control that they carried the double mutation. *huCD34tTA/TetO-H2BGFP* mice (6 females, 11–15 months of age) were divided into two groups, one received tap water *ad libidum*, and the other one received tap water supplemented with Doxy (0.5 mg/mL; Clontech laboratories, Mountain View, CA, USA). After 1-week, the mice in both groups were sacrificed under humane conditions (cervical dislocation previous general anesthesia with an overdose of gaseous isoflurane 4%, Aesica Queenborough Ltd, Queenborough, UK) and their femur harvested for analyses. Selected experiments were performed with 2-months old *huCD34tTA/TetO-H2BGFP* female mice (*n* = 3). All the experiments included single *TetO-H2BGFP* transgenic mice as control for possible leakage of the expression of the transgene. The experiments were performed according to the protocols D9997.121 approved by the Italian Ministry of Health on September 2^nd^ 2021, and according to the European Directive 86/609/EEC.

### 2.2 Flow cytometry determinations

Mononuclear BM cells were incubated with a cocktail of antibodies containing APC-CD117, APC-Cy7-Sca1, PE-Cy7-CD150, biotin-labeled CD48, and biotin-labeled anti-lineage antibodies. After 30 min of incubation on ice, cells were washed and stained with streptavidin-PE-Cy5, and cell fluorescence analyzed with the Gallios FACS analyzer (Beckman Coulter, Brea, CA, USA). The enriched HSC population was defined as LSK (Lin–/CD48–/c-kit+/Sca-1+), while long-term repopulating HSC were defined by the SLAM phenotype (Lin–/CD48–/c-kit+/Sca-1+/CD150+) as previously described (18, 19). All the antibodies were purchased from BD-Pharmingen (San Diego, CA, USA). Dead cells were excluded by Sytox Blue staining (0.002 mM, Molecular Probes, Eugene, OR, USA). Results were analyzed with the Kaluza analysis version 2.1 (Beckman Coulter, Cassina de Pecchi, Italy). Hemi-decrements of the GFP levels expressed by the SLAM were used to divide them into four classes of proliferation as described by Qiu et al. (20). Briefly, cells expressing the maximal level of GFP were defined G0 because did not underwent DNA replication events; cells expressing half of the maximal level were defined G1 because underwent 1 DNA replication cycle; cells expressing a quarter of the maximal level were defined G2 because underwent 2 DNA replication; cells expressing an eighth of the maximal level were defined G3 because underwent 3 DNA replication and, finally cells with barely detected levels of GFP, G4 because they underwent at least 4 DNA replication events. The hemi-decrements described in the paper were determined by hand because of the challenges to properly divide into classes of descendent fluorescence with the FlowJo program (FlowJo™ v10.8, FlowJo LLC, Ashland, Oregon, USA) the signal from the rare SLAM cells (data not shown).

### 2.3 Histology and confocal microscopy determinations

Femurs were fixed in formaldehyde (10%, *v*/*v* with neutral buffer), incubated for 1 h + 4°C with BM biopsy decalcifying solution (EDTA 10%) and included in paraffin. Sections (3 μm) were stained with hematoxylin–eosin (Hematoxylin Cat. #01HEMH2500, Eosin cat#01EOY101000, Histo-Line Laboratories, Milan, Italy). Slides were observed and images acquired with the NanoZoomer 2.0-RS microscope (Hanamatsu Photonic K.K., Hamamatsu City, Japan,), using the NDP.view2 software for NanoZoomer (Hanamatsu Photonic K.K). Sequential sections were stained with DAPI (D9542-5MG, Sigma Aldrich) and analyzed with the confocal microscope Zeiss LSM 900 (Carl Zeiss GmbH, Jena, Germany) in Airyscan mode. Excitation lights were generated by a 405 nm Laser for DAPI and with an argon ion 488 nm laser for GFP. Optical thickness varied from 0.50 μm for the 10x objective to 0.20 μm for the 63x objective. All images were captured under the same conditions and were process and analyzed with the Zen Blue (3.2) software (Carl Zeiss GmbH, November 2021) and the ImageJ (version 1.53t) software (National Institutes of Health http://imagej.nih.gov/ij, accessed on 23 November 2018). Three-dimensional reconstructions were obtained by the full set of stack images, 15 images for the 20 × objective and 34 images for 63 × objective using the Zen Blue software. Nuclei were counterstained with Hoechst 33342, trihydrochloride and trihydrate (Invitrogen), and the samples mounted with Fluor-shield histology mounting medium (Catalog F6182-10MG, Sigma-Aldrich). In selected experiments, the sections were stained with the CD150 antibody (rabbit polyclonal, anti-SLAM/CD150 antibody-N-terminal, cat. no. ab156288, Abcam, Cambridge, UK) coupled with the Alexa Fluor 568-conjugated goat anti-rabbit antibody (Invitrogen, Waltham, MA, USA), as control of the specificity of the GFP label, while endothelial cells were positively identified by staining the sections with the rabbit anti von Willebran factor (vWF) antibody directly conjugated with ALEXAFLUO (Cat. no. ab9378, Abcam, Cambridge, UK).

### 2.4 Single cell quantification of GFP intensity

Images were captured at 8 bit and processed with the Fiji software (ImageJ version 1.53t). The intensity of the GFP signal in the nucleus was measured as described in Supplementary Figure 1. Briefly, (1) color channels are split in 3 single components: Red, Green and Blue. (2) In the Blue channel, it is applied a threshold (60 as lower limit and 255 as highest limit) for selecting the area stained with DAPI (Blue component of the original image) which corresponds to the nucleus. Superimposed or strictly packed nuclei have been resolved applying a binary process called “watershed” that separates adjacent nuclei. (3) Applying this criterium, the number and areas for each single nucleus present in the image is determined. (4) The resulting image is converted into a mask that excluded all pixels outside the holes created by the recognition of the single nuclei. This mask is superimposed to the signal from the Green channel of the same image which contains information on the GFP signal. (5) This process generates an image containing the GFP information (as gray levels) only related to the areas where the nuclei are located. All pixels outside the nuclei are set to zero. For each single nucleus it was then determined the minimum, maximal and Mean value of the gray signal. This process allows to determine for each image the number of nuclei present and the Mean, minimal and maximal level of GFP signal (in arbitrary unit) contained in each nucleus.

### 2.5 Statistical analysis

GFP levels were measured as Mean value by confocal microscopy and as total fluorescence (GFP-A) by flow cytometry. The overall GFP values between untreated and Doxy-treated mice were compared by T Student's Test. Differences in GFP values among untreated and Doxy-treated mice were analyzed by One-way Analysis of variance (ANOVA) and the Tukey-Kramer Adjustment for Multiple Comparisons. Confocal microscopy evaluation of GFP levels in single cells were grouped in classes by two different methods: (1) Levels were grouped in 3 classes according to cumulative percent or (2) grouped in four classes according to the maximum value of GFP intensity registered; the first cut off is represented by half the maximum value of GFP intensity, the second cut off is half the value of the first cut off, and the third cut off correspond to half the value of the second cut off. Chi Square Test was used to compare proportions of the categories of these two grouping classes in untreated vs. Doxy-treated mice and within the bone marrow architecture. Mann-Whitney Test was used to compare fluorescence intensity classes by FACS determination because the data do not show normal distribution. *P*-values for this test were showed both two-tailed and 1-tailed, under the assumption that we expected one group of mice to be better than the other one. All the statistical analysis was performed with the SAS^®^ version 9.4 (SAS Institute Inc. 100 SAS, Campus Drive Cary, NC, USA).

## 3 Results

### 3.1 Tracking HSC cycling by flow cytometry

**Figure 1:**
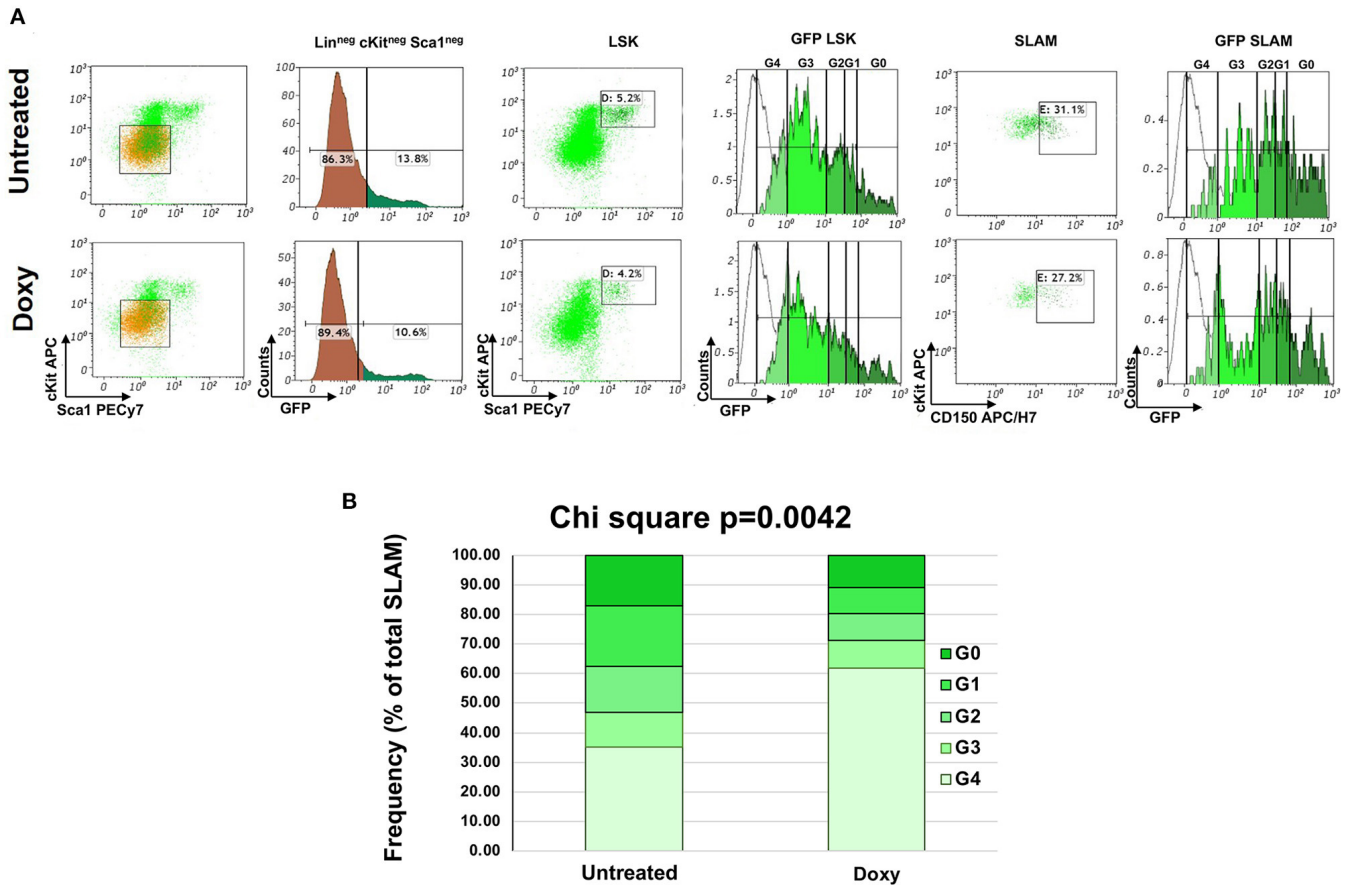
FACS analyses of the hemi-decrement changes in GFP expression occurring in the HSC of the BM after 1 week of exposure to Doxy. **(A)** Representative FACS analyses of Lin-neg BM cells from double mutant mice (15-months old males) either untreated or exposed to Doxy for one week. The levels of GFP expressed by the Lin-neg cells are also presented as control. The levels of GFP expressed by the LSK and SLAM cells are divided into the G0, G1, G2. G3 and G4 gates, as reported (20). The gating used to define the LSK and SLAM populations are also indicated. The white lines indicate the levels of GFP expressed by the BM cells from a single *TetO-H2BGFP* transgenic mouse, used as negative control. The total number of events analyzed is 10,000 per sample which correspond to 500 LSK cells (5% of 10,000 events) and 155 SLAM cells (31% of 500 cells) for the untreated group and to 420 LSK cells (4.2% of 10,000 events) and 114 SLAM cells (27.2% of 500 cells) for the Doxy group. **(B)** Percentage of cells in the various GFP classes over the total frequency of SLAM cells in the BM of untreated or Doxy treated-mice. Data are presented as mean of those observed in three mice per group. The distribution of the cells among the classes in the two groups is statistically significant by Chi-Square test. The statistical analyses of the differences within each class is reported in Supplementary Figure 2.

The Moore laboratory has pioneered the use of untreated and Doxy-treated *huCD34tTA-TetO-H2BGFP* mice to track the cycling of HSC by flow cytometry during the lifespan of mice of C57Bl6 background (15). In preliminary experiments, we tracked the cycling of the HSC in old *huCD34tTA-TetO-H2BGFP* mice harboring the mutation in the CD1 background which, by contrast with C57Bl mice, express a pro-inflammatory phenotype at baseline (26). In these experiments, 15-months old *huCD34tTA-TetO-H2BGFP* mice were exposed to Doxy in their drinking water for only 1 week and the frequency of LSK and SLAM cells, as well as the expression of the GFP in these cells, were evaluated by flow cytometry (Figure 1). Treatment with Doxy did not alter the frequency of LSK or SLAM cells which were equivalent in the two groups (LSK: 5.4 ± 0.5 vs. 5.1 ± 1.6% of total Lin^neg^ cells; SLAM: 34.5 ± 4.4 vs. 32.15.4 ± 7.2 of the LSK, respectively). GFP was expressed almost exclusively by the LSK and SLAM. Very few (~10%) of the Lin^neg^ cells contained GFP. In a previous study, we identified that the Lin^neg^cKit^neg^Sca1^neg^ cells that express GFP are larger than the other cells, contain the GFP signal mostly in their cytoplasm and express F40/80, suggesting that they are macrophages that have phagocytized HSC (26). By contrast, robust numbers of LSK and SLAM expressed GFP at a level which spanned over 4 logs, supporting the knowledge that expression of *TetO-H2BGFP* is switched off in the progeny generated by the first differentiative division of the HSC and that its content decreases by half with every cell division thereafter (20). Treatment with Doxy for only 1 week induced significant changes in the distribution of the SLAM among the various classes of fluorescence intensity with an enrichment in the frequency of SLAM expressing barely detectable levels of GFP (Figure 1). In depth analyses of the differences in the distribution of the SLAM in the various classes of GFP intensity indicates a statistically significant reduction in the frequency of SLAM in the G1 and G2 classes and a statistically increase in that of SLAM in G4 after Doxy treatment by 1-tailed Mann-Whitmey analyses (Supplementary Figure 2).

The levels of cKIT and CD150 expressed by SLAM in the untreated and Doxy group according to their fluorescence classification was also determined (Table 1). There is no difference in the level of expression of these two antigens among the classes within the same experimental group. However, the G1 and G2 cells from the untreated group expressed levels of cKIT significantly greater than those of the corresponding cells from the Doxy group, while the G0 and G1 cells of this group express levels of CD150 significantly lower than those expressed by the corresponding cells in the Doxy group. The physiological significance of these differences is presently unclear.

**Table 1:** Comparison of the levels of cKIT and CD150 (as mean fluorescent intensity, MFI) expressed by the LSK and SLAM cells divided for fluorescence classes presented in Figure 1.

	**LSK**			**SLAM**		
	**Untreated (*****n*** = **3)**	**Doxy (*****n*** = **5)**	***P*** **-value (** ***t*** **-test)**	**Untreated (*****n*** = **3)**	**Doxy (*****n*** = **5)**	***P*** **-value (** ***t*** **-test)**
**cKIT (CD117) MFI**						
G0	26.5 ± 1.4	25.8 ± 4.0	0.5354	24.0 ± 2.2	24.9 ± 3.9	0.7738
G1	28.7 ± 3.5	25.9 ± 1.5	0.1192	28.9 ± 2.5	24.4 ± 2.9	0.0087
G2	32.8 ± 4.2	28.0 ± 2.0	0.0480	36.6 ± 6.8	28.6 ± 2.4	0.0300
G3	30.2 ± 5.0	27.0 ± 3.1	0.2688	29.9 ± 18.2	29.8 ± 5.1	0.9915
G4	24.6 ± 1.9	24.1 ± 3.8	0.8158	18.1 ± 5.5	23.2 ± 3.0	0.1092
**CD150 MFI**						
G0	n.a.	n.a.		28.4 ± 3.3	47.8 ± 9.6	0.0128
G1	n.a.	n.a.		26.5 ± 3.0	41.2 ± 6.9	0.0101
G2	n.a.	n.a.		22.1 ± 4.2	38.2 ± 11.3	0.0537
G3	n.a.	n.a.		20.5 ± 5.2	35.3 ± 12.3	0.0875
G4	n.a.	n.a.		26.5 ± 10.4	35.6 ± 12.9	0.3268

n, number of mice in the experimental group; n.a., not applicable.

These results indicate that the SLAM in the BM of old mice expressing constitutively higher levels of TGF-β are a dynamic cell population with few of the very positive cells entering in cycle but with as many as 12–7% of the intermediate positive cells entering cycle over a 1-week period.

### 3.2 Tracking HSC cycling by confocal microscopy

**Figure 2:**
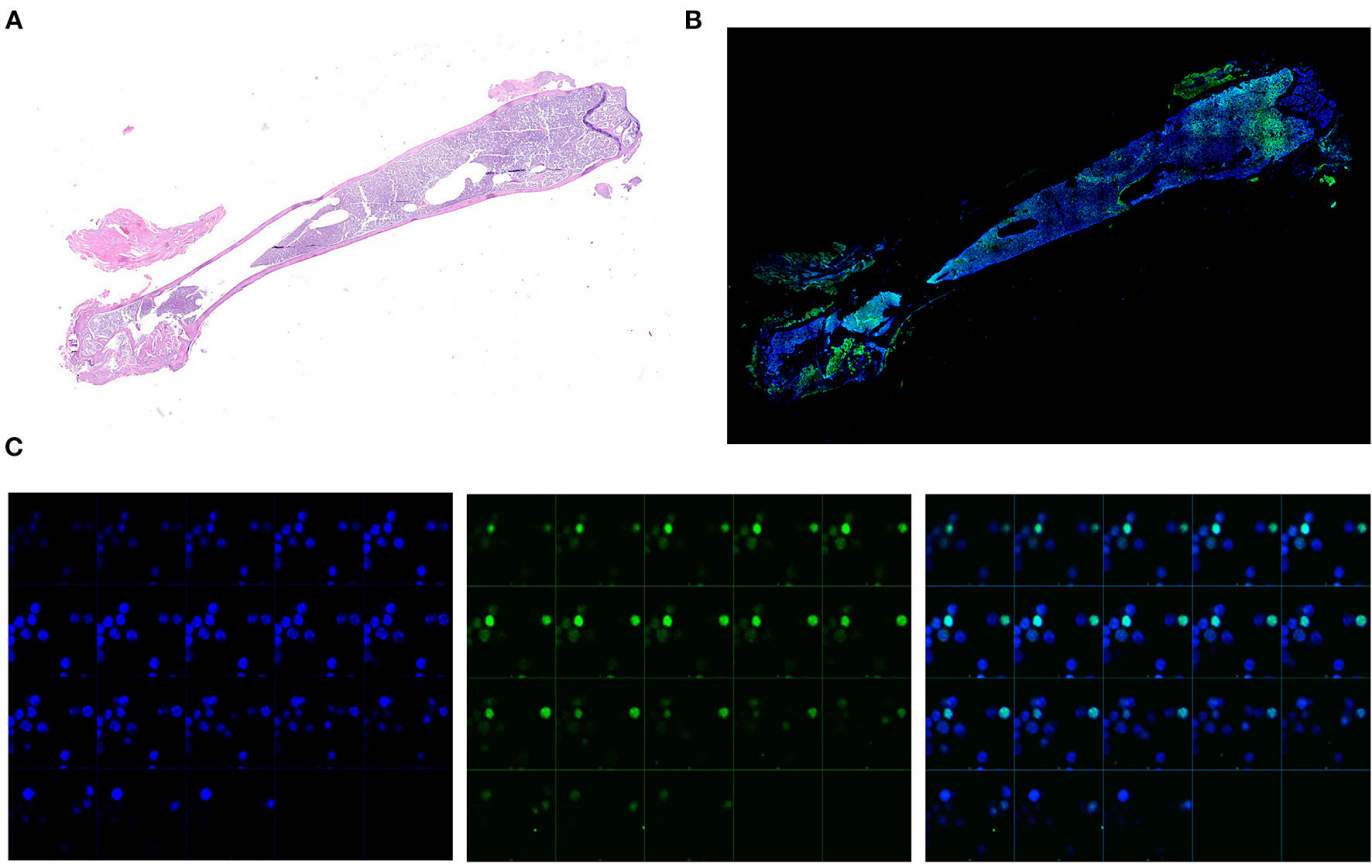
Representative analyses of the GFP levels expressed by the single cells from the BM of an untreated double mutant female mouse (11-months old). **(A)** Reconstruction of the whole femur from the representative double mutant mouse stained by Hematoxylin/Eosin. Scanning was performed with a resolution equivalent to that of a x40 objective. **(B)** Reconstruction of the whole femur from the same representative double mutant mouse shown in A stained with DAPI by confocal microscopy. The original images were acquired at x200 magnification. Blu = DAPI, Green = GFP. **(C)** Zeta stacking (each photogram a different focal plane) at X400 magnification of images collected by confocal microscopy in order to confirm the nuclear colocalization of both the DAPI and the GFP signal. Two representative GFP positive cells are shown. Images are presented as individual DAPI (left panels) and GFP (central panels) and as merged (right panels) signals.

To investigate the distribution of GFP positive cells within the BM architecture, we first confirmed that great numbers of small cells expressing robust levels of GFP signal in their nucleus were detectable by confocal microscopy (Figures 2A, B). By contrast, GFP positive cells were not detectable in the femur from a single *TetO-H2BGFP* mouse, used as negative control (Supplementary Figure 3). Sequential Z stacking images indicated that the green GFP signal was co-localized with DAPI, confirming that GFP was localized in the nucleus of these cells (Figure 2C).

**Figure 3:**
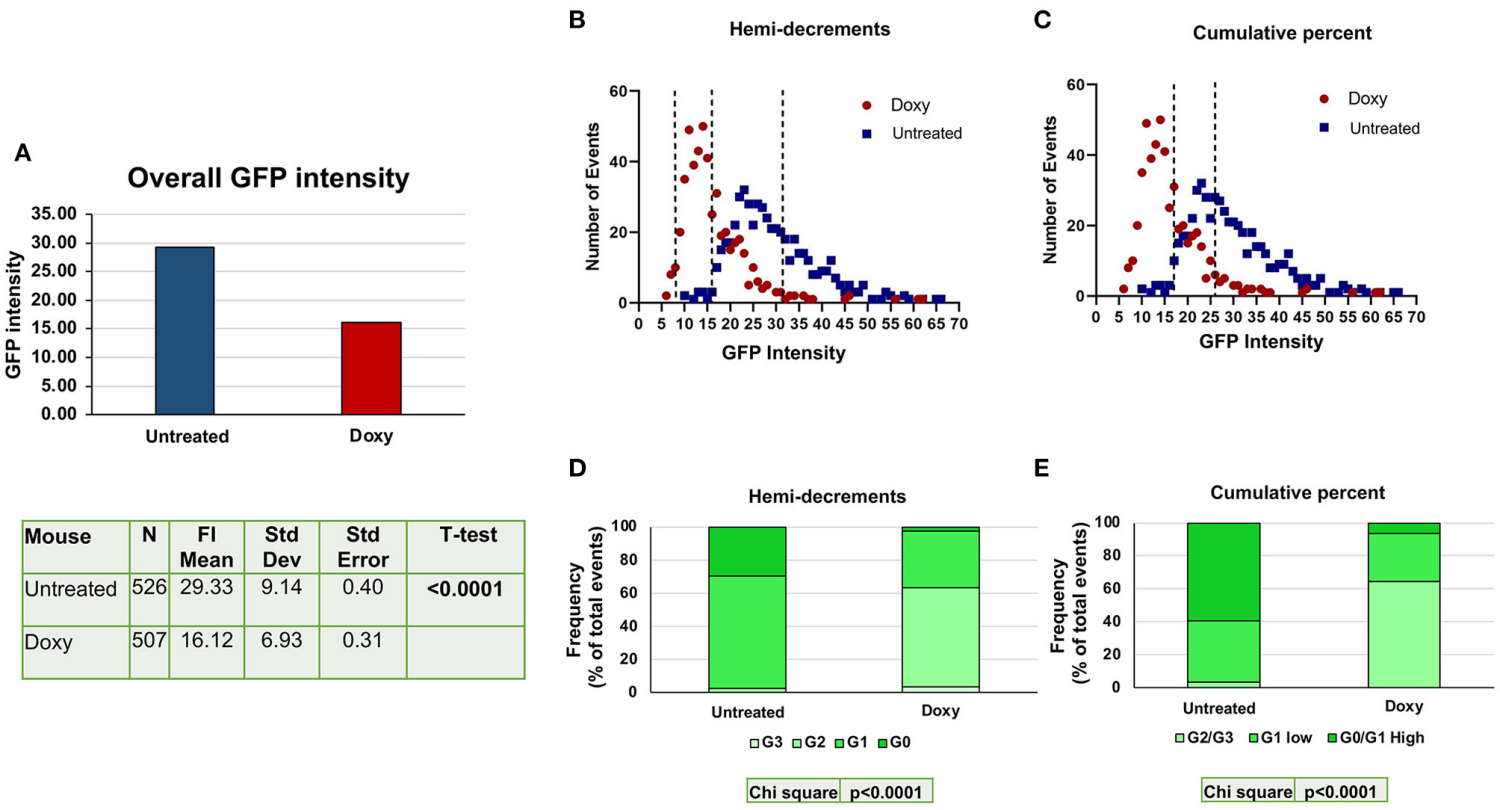
Doxy reduces the levels of GFP expressed by the single cells detected by confocal microscopy in the femur sections from untreated or Doxy-treated mice. **(A)** Comparison between the overall GFP levels expressed by single cells from the BM sections of the untreated (blue) and Doxy-treated (red) mice (two mice per group), as indicated. All the GFPpos cells present in one section were considered. The data are reported with statistical analyses in a table format below the graph. **(B)** Distribution of the GFP levels expressed by individual cells (each dot a different cell) from the femur of untreated (blue dots) and Doxy-treated (red dots) mice, as indicated. In this case, the GFP levels are divided into four classes by hemi-decrement analyses of the maximal levels of GFP intensity determined. **(C)** The same distribution of the GFP levels shown in B but divided into 3 classes according to the cumulative percentage of the values distribution. The total number of events presented in **(B, C)** is 1,033 (526 for untreated and 507 for the Doxy-treated group). **(D, E)** Frequency of the cells in the various GFP classes over the total frequency of SLAM cells observed in the BM of either untreated or treated-mice. Data are presented as mean of those observed with two mice per group. Both in **(D, E)**, differences in cell distribution among the classes in the two groups are statistically significant by Chi-Square test.

Using the criteria defined in Supplementary Figure 1, the levels of GFP detected in the nucleus of single cells from the BM of untreated and Doxy-treated double mutant mice were determined (>500 cells per group) (Figure 3). Overall, the levels of GFP intensity expressed by the single cells from the Doxy-treated mice was significantly lower than that expressed by the untreated mice (Figure 3A). As observed by flow cytometry, the levels of GFP determined by confocal microscopy in the single cells from both groups did not have a normal distribution and showed a peak at 23 and 14 arbitrary units, respectively. Since the GFP is expressed in the nuclei only of the HSC and of their immediate progeny, we used GFP staining and small size as criteria to recognize HSC and their immediate progeny. We divided the cells according to their GFP intensity by two statistical methods: the first method was similar to that used by flow cytometry and divided the cells into four classes based on hemy-decrements of GFP intensity (Figure 3B). These classes are basically similar to the G0 (never divided); G1 (divided once), G2 (divided twice) and G3 (divided three times) recognized by flowcytometry. It is worthy of attention that, since the confocal analysis used in this study did not include a positive marker for HSC identification, it does not allow to identify the progeny of the fourth division of HSC (G4, that express barely detectable levels of GFP by flow cytometry). The second method divides the cells into cumulative percentage of their intensity score in three classes which partially overlap with those identifies by hemi-decrements. In both cases, the difference in the distribution of the individual cells among the classes of GFP intensities from the untreated and the Doxy-treated mice is highly significant (*p* < 0.0001 by Chi square) (Figures 3D, E). Also by confocal microscopy, the greater differences between untreated and Doxy-treated cells were represented by a decrease in the frequency of cells expressing the highest levels and increases in the cells expressing intermediate levels of GFP.

### 3.3 The most positive GFP+ cells are localized around vessels

**Figure 4:**
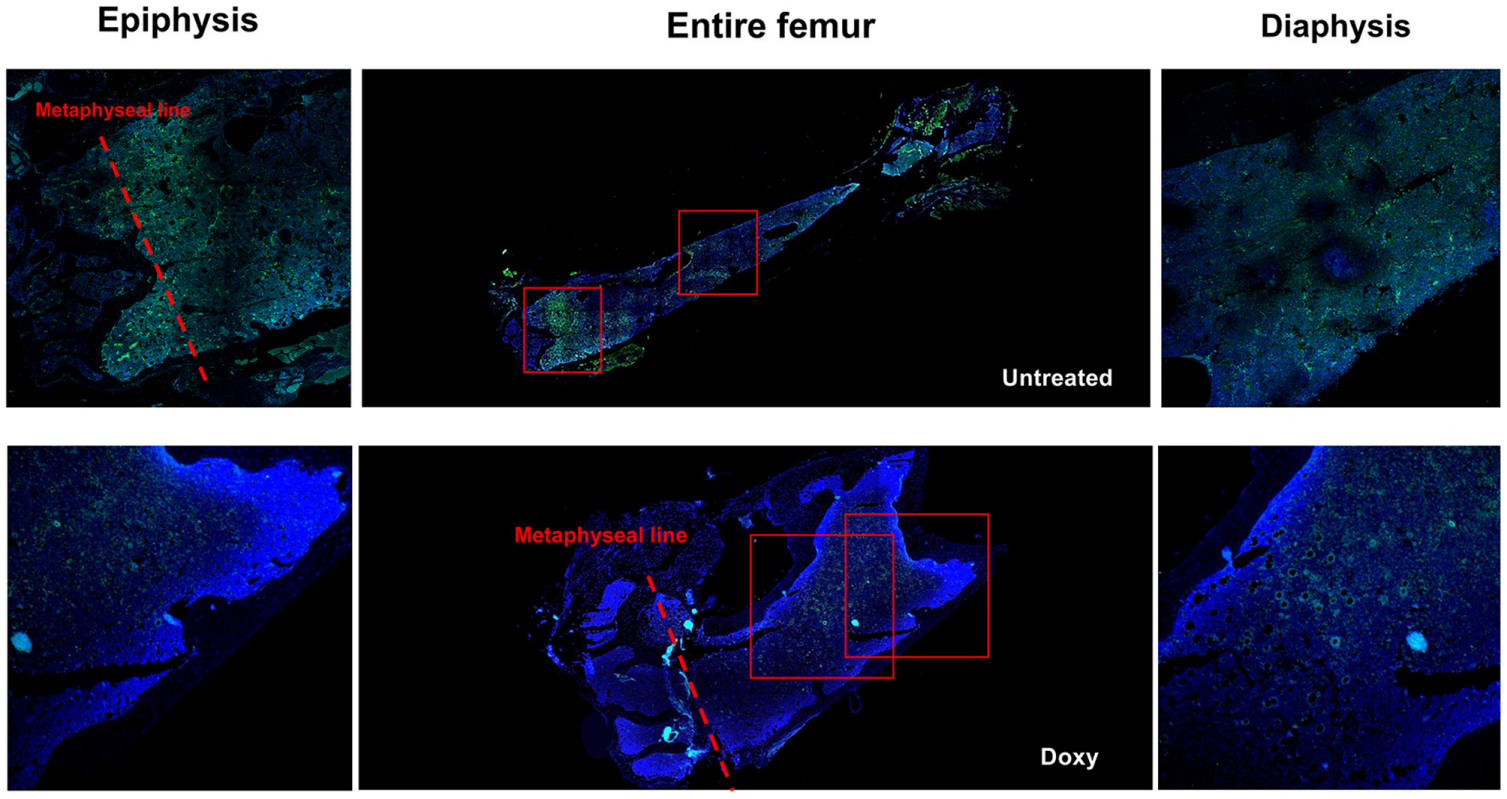
Both in untreated and in Doxy-treated mice, GFP positive cells are localized in all the areas of a femur (both in the epiphysis and in the diaphysis). Reconstruction of the femur from a representative untreated and Doxy-treated mutant mouse **(middle panels)** and detail of the epiphysis **(left panels)** and of the diaphysis **(right panels)** of the same femur obtained by confocal microscopy. The Dapi and GFP signals are merged. The rectangles in the middle panels indicate the areas shown at greater magnification in the panels on the side. Original magnification x100 and x200 for the entire femurs and their detail, respectively.

**Figure 5:**
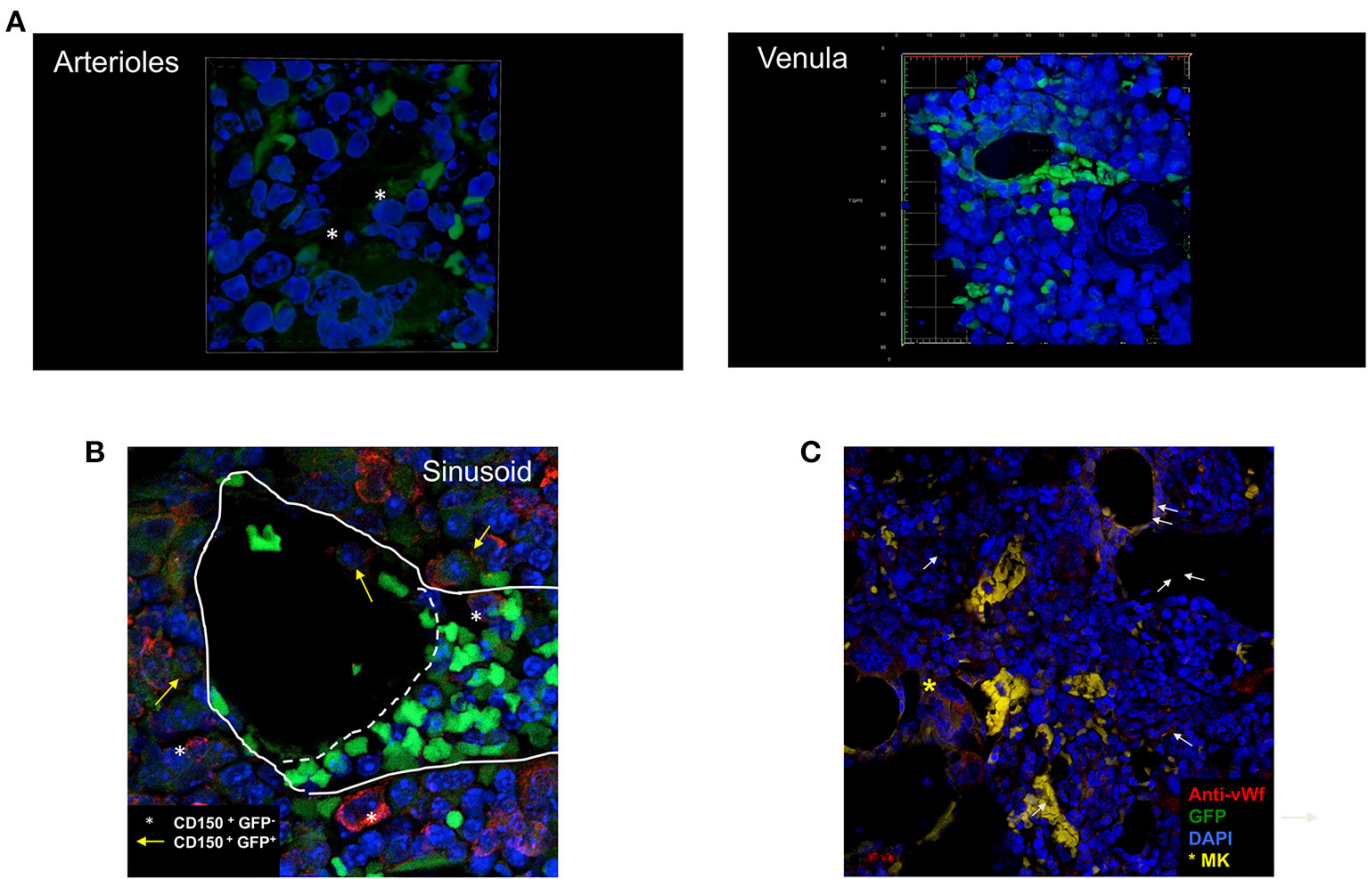
Cells expressing high levels of GFP are often found located around vessels. **(A)** 3D reconstruction of an arteriole (top panel) and of a venula (bottom panel) surrounded by small GFP positive cells. **(B)** Confocal microscopy observation of a representative BM section stained with the von Willebrand (vWF) antibody. The arrows indicate representative GFP pos cells localized around the vessels which are lined by vWF positive cells. To be noted the presence of a vessel surrounded by vWF positive endothelial cells which does not contain GFP-positive cells (symbol) but associated with a megakaryocyte in the process to release platelets (the large cells with multi-lobated nucleus positive for vWF indicated by the asterisk). **(C)** Confocal microscopy observation of a representative BM section stained with the CD150 antibody showing that all the GFPpos cells around the vessel are also CD150pos (yellow arrows) while there are same CD150pos which are not labeled by GFP (asterisks). The image shows the anastomosis between two venules, one cut transversally and another one cut longitudinally. This anastomosis identifies the two vessels as sinusoids. The straight line indicates the localization of the basal lamina while the dotted line indicates the junction between the two vessels. The small green cells in the vessel cut longitudinally are red blood cells. To be noted the GFPposCD150+ cells located in the lumen of the vessel cut transversally and the GFPnegCD150pos cells located near the transversal vessel that appears in cytodieresis. Original magnification x600 (arterioles and venula) and x200 in all the other panels.

By confocal microscopy analysis, the cells expressing GFP were distributed along the entire femur (Figure 4). Both in the untreated and in the Doxy-treated mice, the GFP positive cells were more frequent in the epiphysis below the metaphyseal line and in the medulla. They were localized either around optical empty spaces, or in their proximity (within 20 μm) although some GFP positive cells was also observed within the BM parenchyma. Observations at greater magnification, indicated that the empty spaces which were lined by strongly GFP positive cells had the structure of small vessels (Figure 5A). Given the thickness of the sections (3 μm) and the fact that we have not made 3D BM reconstructions by combining images collected from multiple consecutive sections, the distinction between cells located near vessels and within the parenchyma may be artificial.

By morphology, we identified two types of vessels: arterioles, circular areas well delimitated by a lumen wall composed by elongated cells with endothelial-like morphology and surrounded by a thick layer of cells, probably representing muscle cells, and venules, elliptic/collapsed spaces surrounded by a thin layer of elongated cells. In most of the cases, the morphology does not allow us to discriminate whether these elliptic structures are represented by veins or sinusoids. The morphological hypothesis that these structures are vessels was confirmed by determining that they were lined by cells that reacted with a von Willebrand Factor (vWF) antibody, a marker for endothelial cells (Figure 5C).

To confirm that the GFP labeled cells surrounding the vessels are HSC, we performed a pilot experiment in which the BM sections were stained with the CD150 antibody (the SLAM marker used in flow cytometry) (Figure 5B). This experiment confirmed that all the GFP labeled cells express CD150, and are therefore HSC, and identified a CD150pos cell population which is not labeled by GFP and that may represent the G4 Class identified by flow cytometry.

**Figure 6:**
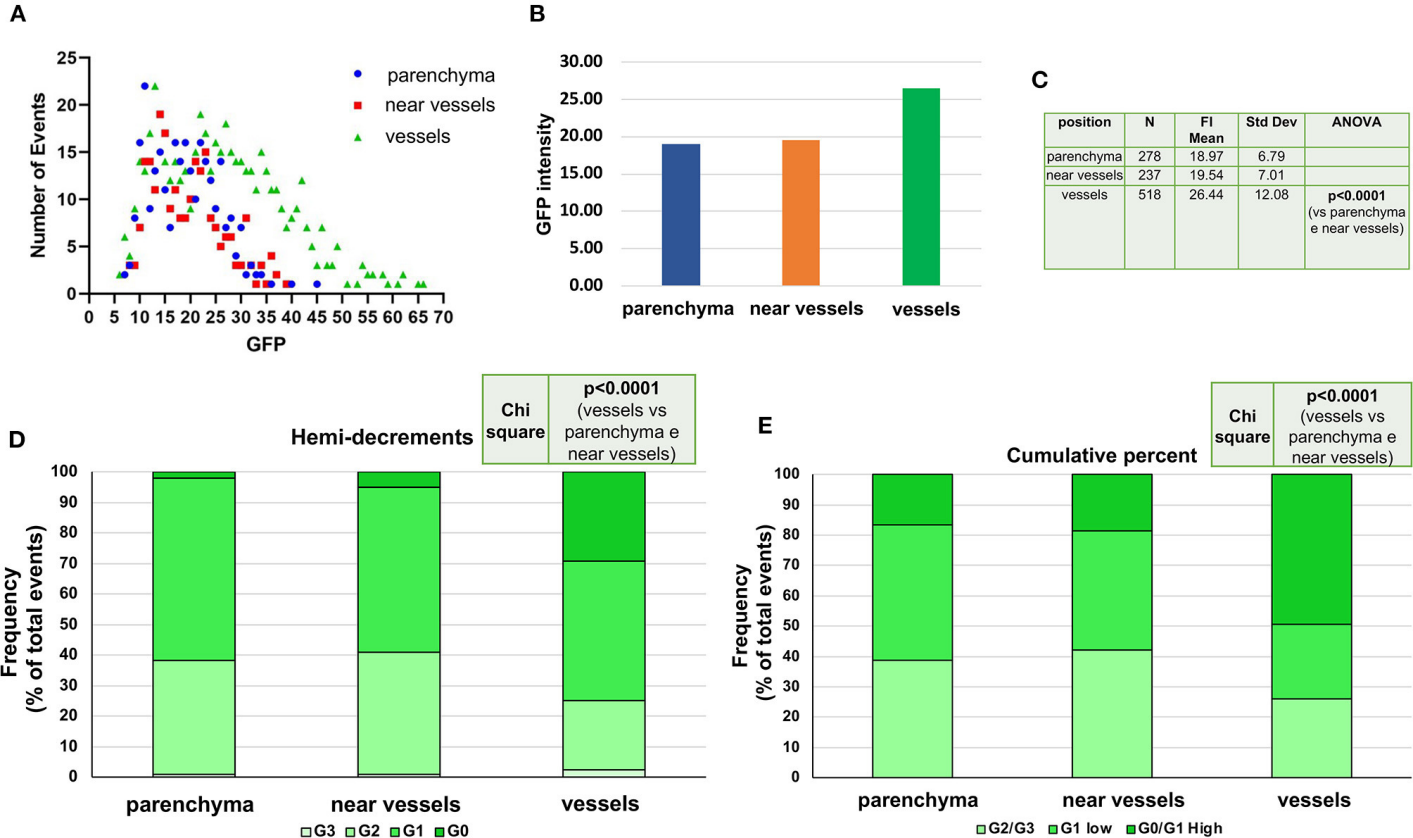
The cells expressing the highest levels of GFP are localized in proximity of the vessels. **(A)** Distribution of the levels of GFP expressed by single cells (each symbol a different cell) according to their localization. GFP positive cells were divided into cells surrounding vessels, in close proximity (within 20 ?m) of a vessel or within the BM parenchyma, as indicated. The cell localization was determined by the Image J program and by the control software of acquisition ZenBlue. **(B)** Overall GFP intensity expressed by the cells according to their location. **(C)** Number and GFP content [Mean (+/-SD)] of the single GFP+ cells analyzed for each localization. The p value was analyzed by ANOVA. **(D, E)** Distribution of single cells expressing different levels of GFP fluorescence intensity divided by either the hemi-decrement or the cumulative percent criteria (the same criteria as in Figure 3). In both analyses, Chi-Square test shows statistically significant difference in the localization of the GFP positive cells, with the cells expressing the highest levels localized around vessels.

Single cell analyses of the levels of GFP expression according to location confirmed that, overall, the cells expressing GFP were mostly located around vessels which were surrounded by almost 50% of all the GFP+ cells detected in a femur section (518 vs. 237 + 238 cells, respectively, Figures 6A, B). It also indicated that 30% (by hemi-decrements) or 70% (by cumulative percentage) of the cells with the highest levels of GFP expression were located near vessels (*p* < 0.0001 in both cases) (Figures 6C–E). As expected, in old mice, very few GFP labeled cells were present near the endosteum of the trabecular bone (Supplementary Figure 4).

To start investigated the effects of age on the frequency and localization of GFP+ cells, a preliminary experiment was performed to analyze the distribution of GFPpos cells within the bone marrow architecture of young (2–3-months old) mice (Supplementary Figure 5). The frequency of GFP+ cells in the BM of these young mice was much lower than that detected in the BM of old mice (compare the frequency of GFP+ events in Figure 4; Supplementary Figure 5). In addition, the GFP+ cells were localized both near the endosteum of the bone and the vessels.

These results confirm that in old mice the endothelial cells surrounding the vessels are an important niche for the most primitive HSC.

### 3.4 Doxy-treatment spares the GFP label expressed by cells surrounding the arterioles

**Figure 7:**
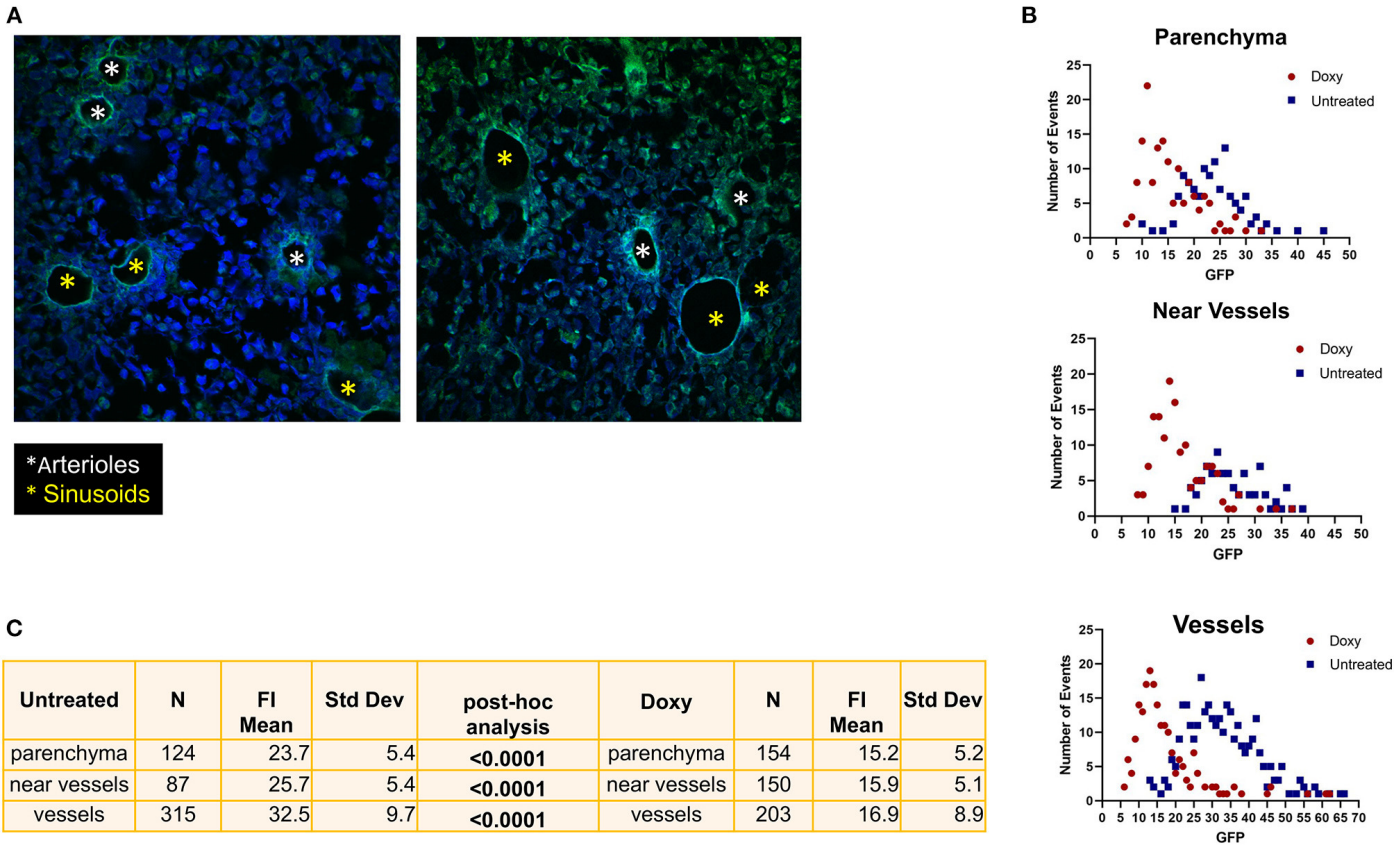
Treatment with Doxy specifically reduces the GFP levels expressed by cells surrounding venules. **(A)** Confocal microscopy analyses of the femur from representative untreated and Doxy-treated mice stained with DAPI and GFP. The images indicate that in untreated mice cells expressing high levels of GFP surround both arterioles (white asterisks) and venules (yellow asterisks) while in the Doxy-treated animal high GFP expression is retained only by cells surrounding the arterioles. **(B)** Distribution of the GFP fluorescence intensity expressed by single cells from the BM of untreated (blue dots) or Doxy-treated (red dots) mice (two mice per group) according to their localization within the BM architecture (each symbol a different cell). **(C)** Number of single cells analyzed and Mean (+/−SD) of the levels of GFP expressed by the single cells from the BM of untreated or Doxy-treated mice according to their localization. The *p*-values were calculated by ANOVA with a *post-hoc* analysis.

In untreated mice, the strongest GFP positive cells were observed surrounding vessels with the morphology of both arterioles and venules (Figure 7A). One week of Doxy treatment reduced the levels of GFP expressed by the cells located in all the areas of the femur (Figures 7B, C). The greatest reductions, however, were observed in the levels of GFP expressed by the cells surrounding the vessels (50% overall reduction from 32.5 vs. 16.9 fluorescence intensity, before and after Doxy, respectively). Morphological analyses indicates that the fluorescence intensity is either lost or retained by all the cells surrounding a specific vessel (Figure 7A). In the Doxy-treated group, the cells that retain the highest levels of GFP appear co-localized all around vessels with the morphology of arterioles.

## 4 Discussion

The double *huCD34tTA-TetO-H2BGFP* mutant mouse has provided important information on the dynamics of HSC cycling over time (15, 21, 23). These studies have identified that 60% of HSC from young (12-weeks old) C57Bl mice remain quiescent over a period of 12 weeks (20). In this model, HSC are supposed to be retained into quiescence by the high levels of expression of genes of the TGF-β pathway. Treatment with factors, like G-CSF, which induce HSC mobilization, does not reduce the number of quiescent HSC, suggesting that in these mice HSC have greater affinity for the niche that retain them quiescent (23). With age, HSC enter into a state of dormancy which is retained for the rest of the life of a mouse (15). Whether the niche which sustains the “dormant” HSC in old mice is similar from that that sustain “cycling” HSC in young mice has not been clarified as yet.

Other studies, using multi-color confocal microscopy and expression profiling, have identified that in adult mice HSC are preferably located near vessels and are retained into quiescence by the niche which surrounds the arterioles while they are induced in proliferation by that surrounding the venules (1, 2, 4, 6, 29, 30). Since in old mice the HSC which remain quiescent are a minority, most of the HSC should be associated with a niche still poorly identified that sustain their proliferation, causing the HSC exhaustion which results in the anemia and thrombocytopenia observed in old mice.

The study presented here couples the power provided by the double *huCD34tTA-TetO-H2BGFP* mutant mouse with that of confocal microscopy to study the association of the HSC with their niche as they cycle in old mice with the spontaneous pro-inflammatory profiling provided by the CD1 background (28). In fact, compared to mice of other strains, such as C57Bl mice, the pro-inflammatory profile of the bone marrow from CD1 mice includes increased bioavailability of TGF-β, a factor known to induce HSC into quiescence promoting their self-replication (24).

A first set of experiments validated information on HSC cycling obtained in our model by comparing the levels of GFP intensity observed in the SLAM by flow cytometry with that obtained by measuring the nuclear levels of GFP in cells with a small size (a morphological criteria for HSC identification) by confocal microscopy. The fact that GFP signaling was barely observed in the BM of single *TetO-H2BGFP* mutant mouse with both methods, provided evidence for the specificity of our determinations. A significant difference between determinations of the cycling status of the HSC by flow cytometry and by confocal microscopy is that, by flow cytometry HSC are independently recognized by the SLAM marker while by the confocal microscopy assessment used in this paper GFP is both a marker for the HSC and for their cycling state. For this reason, HSC that divided more than four times, i.e., expressing barely detectable levels of GFP, can be identified by flow but not by the confocal microscopy method used in this manuscript. With this caveat, there was a good correlation between the HSC which never divided (quiescent HSC) and those that underwent 1, 2, and 3 divisions over one 1-week period determined with the two methods. In fact, with both methods, only a minority (10 vs. 5–10%) of the cells remained quiescent over a 1-week period while most of them (60% in both cases) underwent 1–3 cell divisions. These results indicate that in old mice HSC are a very dynamic populations and undergo numerous divisions over just 1 week time.

Single cell analyses of the distribution of the labeled HSC within the BM microenvironment indicated that most of the labeled HSC were located near vessels. It also indicated that the cells surrounding the vessels were those expressing the highest GFP intensity. This last result indicates that the HSC near the vessels are either quiescent or had divided at most once. After Doxy, the majority of the GFP labeled cells were still located near vessels. However, there was no longer difference between the intensity of GFP expressed by the HSC near the venules and those present in the BM parenchyma, indicating that in just 1-week period the HSC around the vessels had cycled few times. Interestingly, the arterioles were the vessels which were surrounded by the few HSC which retained label after Doxy, suggesting that this niche supports either HSC which are quiescent or that enter in cycle at a rate lower than once a week. It is also possible that this niche sustains the dormant HSC identified by the Moore laboratory to be uniquely present in old mice (15). Since, because of the pro-inflammatory environment provided by the CD1 background, the levels of TGF-β are similarly high around the arterioles and the venules, it is possible that the arterioles produce additional factors, still to be identify, which retain HSC into a quiescent/dormant state (7). This hypothesis is supported by the observation that all the cells surrounding a specific arteriole retain label. However, further confocal microscopy studies, outside the purpose of this paper, which will compare the location of the labeled HSC using age, sex and time of Doxy treatment as independent variables, are necessary to clarify whether in old mice the HSC around the arterioles are quiescent or dormant. The rigor of these new studies will be greatly increased by including the CD150 marker to positively identify the HSC.

In conclusion, our results indicate that aged HSC are actively cycling and are mostly associated with venules, confirming that these cells are biased for interacting with the niche that instructs them to differentiate (4, 7, 30). The very few old HSC that retain label are located around the arterioles, providing morphological evidence that this niche may represent the niche that sustain the dormant HSC present in old mice.

## Data availability statement

The original contributions presented in the study are included in the article/Supplementary material, further inquiries can be directed to AM, amigliaccio@altius.org.

## Ethics statement

The experiments were performed according to the protocols D9997.121 approved by the Italian Ministry of Health on September 2nd 2021, and according to the European Directive 86/609/EEC.

## Author contributions

MM and FA performed all the experiments. OP performed the statistical analyses of the data. PV, FM, and MV maintained the mouse colony, treated the mice, and performed flow cytometry determinations. ARM, MF, and MZ designed the study, interpreted the data, and wrote the manuscript. All authors read the manuscript, concur with its content, contributed to the article, and approved the submitted version.

## Supplementary material

Supplementary Figure 1 Graphic representation of the method developed to quantify the levels of GFP in the nucleus of single cells in the BM from the double mutant mice. **(A)** Workflow describing the process of quantification of the GFP intensity in single cells from the femur of the mice. The first step is the reconstruction of the image of the entire femur by slide scanner for H&E or by stitching the individual images acquired by confocal microscopy stained with DAPI. In both cases images have the same resolution as that acquired by a 20X objective. The second step consists in the examination of the green signals released by the GFP acquired by confocal microscopy. The third step is the quantification of the GFP intensity in the single cells by ImageJ (see Material & Methods for further detail of this quantification). **(B)** Visualization of the sequential steps described in A including representative images. Images are acquired with a 40x objective. The first step is to split color channels and analyze the Blue component converting them in a 8 bit, 255 gray levels image. Same threshold values have been adopted for all images. The resulting image is then used as mask to contour of the objects of interest in the Green component image in which the gray levels have been measured.

Supplementary Figure 2 Doxy significantly decreases the frequency of GFP positive SLAM in the G1 and G2 group and increases those present in the G4 group detected by flow cytometry. Data are presented as median (plus min and max) of those observed with three mice per group. Statistical analyses was performed by Mann-Whitney and by Mann-Whitney (1-tailed) tests.

Supplementary Figure 3 GFP is not detected by confocal microscopy in the BM from mice arboring only the *TetOH2BGFP* transgene. **(A)** Reconstruction of the whole femur from a representative single *TetOH2BGFP* transgenic mouse stained either by Hematoxylin/Eosin **(A)** or analyzed by confocal microscopy for DAPI and GFP. Original magnification x200.

Supplementary Figure 4 GFP labels cells are seldomly found close to the endosteum of the trabecular bone. Bright field **(on the left)** and GFP/Hoechst staining **(on the right)** of a section of the trabecular bone from a representative 15-months *huCD34tTA/TetO-H2BGFP* transgenic male mouse. The dotted line in the left panel indicates the area occupied by the trabecular bone. Original magnification x600.

Supplementary Figure 5 Distribution of the GFP label cells within the bone architecture of a young (2–3-months old) *huCD34tTA/TetO-H2BGFP* transgenic male mouse. **(A)** Reconstruction of the whole femur from a 2-months double mutant mouse stained by Hematoxylin/Eosin. Scanning was performed with a resolution equivalent to that of a 20x objective. **(B)** Reconstruction of the whole femur from the same representative double mutant mouse shown in A stained with DAPI by confocal microscopy. **(C)** Large magnifications of the areas of the epiphysis, diaphysis and trabecular bones indicated in **(B)** showing the detail of the localization of the GFP labeled cells within the bone architecture. Results are representative of those observed with three individual mice. Original magnification x40 and x200 for the entire femur and its details, respectively.
