# Supplementary figures and images for: Single cell analysis of the localization of the hematopoietic stem cells within the bone marrow architecture identifies niche-specific proliferation dynamics
Source: Front Med (Lausanne). 2023 Apr 28;10:1166758. doi: 10.3389/fmed.2023.1166758 (PMC10175646; doi:10.3389/fmed.2023.1166758)

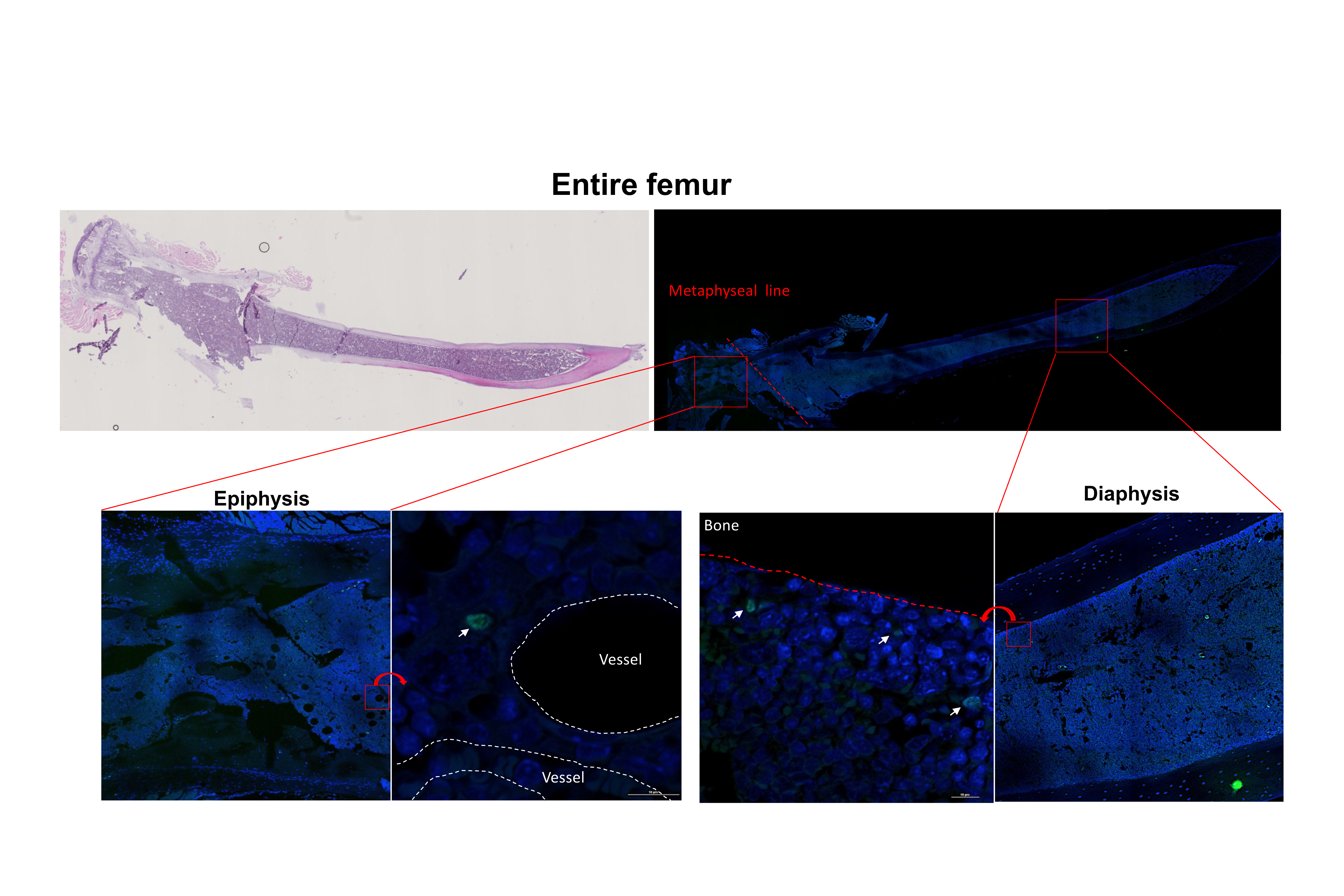

Supplement: Supplementary Figure 5 — Distribution of the GFP label cells within the bone architecture of a young (2–3-months old) huCD34tTA/TetO-H2BGFP transgenic male mouse. (A) Reconstruction of the whole femur from a 2-months double mutant mouse stained by Hematoxylin/Eosin. Scanning was performed with a resolution equivalent to that of a 20x objective. (B) Reconstruction of the whole femur from the same representative double mutant mouse shown in A stained with DAPI by confocal microscopy. (C) Large magnifications of the areas of the epiphysis, diaphysis and trabecular bones indicated in (B) showing the detail of the localization of the GFP labeled cells within the bone architecture. Results are representative of those observed with three individual mice. Original magnification x40 and x200 for the entire femur and its details, respectively. [file Image_5.JPEG]
